# Extramedullary hematopoiesis in the absence of myeloproliferative neoplasm: Mayo Clinic case series of 309 patients

**DOI:** 10.1038/s41408-018-0156-6

**Published:** 2018-11-19

**Authors:** N. Fan, S. Lavu, C. A. Hanson, A. Tefferi

**Affiliations:** 1 Division of Hematology, Department of Medicine, Mayo Clinic, Rochester, MN USA; 2 Division of Hematopathology, Department of Laboratory Medicine, Mayo Clinic, Rochester, MN USA

Extramedullary hematopoiesis (EMH) implies the production of erythroid and myeloid progenitor cells outside of the bone marrow. EMH in adults is typically seen in patients with myeloproliferative neoplasms (MPNs) but its association also with other conditions, including thalassemia, has long been recognized^1^. In both MPN and non-MPN settings, the liver and spleen are the two most frequent sites of EMH and it has been hypothesized that circulating hematopoietic cell filtration (entrapment), possibly via endothelial cell expressed ligands, such as chemokine ligand 12, rather than splenic stroma account for the particular phenomenon^2–4^; the concordant detection of MPN-specific mutations, such as *JAK2*V617F, and specific cytogenetic abnormalities in both bone marrow and splenic tissue of affected patients, supports this contention^5,6^.

Circulating hematopoietic progenitors mobilized as a result of otherwise nonspecific hematopoietic stressors have also been implicated in seeding non-hepatosplenic EMH (NHS-EMH)^7^. The latter has been reported in a variety of organs, including the central nervous system^8^, ovaries and tubes^9^, the skin^10^, the lungs and pleura^11^, the pericardium^12,13^, lymph nodes^14^, and other sites. In a previously published report of 27 Mayo Clinic cases of NHS-EMH diagnosed antemortem between 1975 and 2002^15^, the most common associated condition was myelofibrosis and the most frequent involved site the thoracic vertebral column. The current study focuses on the Mayo Clinic experience with EMH cited in the absence of MPN. The objectives were to systematically describe associated conditions and involved sites and identify “idiopathic” cases and review their management and long-term outcome.

After approval by the Mayo Clinic institutional review board, institutional databases were screened through the Mayo Clinic Advanced Cohort Explorer (ACE) Tool, in order to identify patients with EMH. ACE is a clinical data repository maintained by the Unified Data Platform; ACE is enriched with multiple source patient information including patient demographics, diagnosis, hospital notes, laboratory reports, flowsheets, pathology reports, and clinical notes. With ACE’s text search functionality, we queried “extramedullary hematopoiesis” or “EMH”. We performed a retrospective database review of all identified patients between 1975 and 2018. Demographic, biochemical, genetic, radiological, and pathological data were collected and reviewed.

ACE identified 1933 cases of “EMH”. Extensive review confirmed the absence of associated MPN in 336 cases. Among these, 27 cases involved pathology remarks during tissue biopsy for liver transplant and were excluded from further analysis. The most frequent associated conditions in the remaining 309 cases (Table 1) included myelodysplastic syndromes (MDS) (*n* = 41; 13%); acute myeloid leukemia (AML) (*n* = 28; 9%); hemolytic anemia (*n* = 24; 8%); thalassemia (*n* = 22; 7%); non-Hodgkin’s lymphoma (NHL), with excess cases with splenic marginal zone lymphoma (*n* = 19; 6%); immune thrombocytopenic purpura (ITP) (*n* = 17; 6%); metastatic cancer, with breast cancer being the most frequent (*n* = 17; 6%); plasma cell neoplasms, including polyneuropathy, organomegaly, endocrinopathy, monoclonal protein, and skin changes (*n* = 12; 4%); hereditary spherocytosis (*n* = 8; 3%); cirrhosis (*n* = 7; 2%); acute lymphoblastic leukemia (*n* = 6; 2%); chronic lymphocytic leukemia (*n* = 6; 2%); Hodgkin’s lymphoma (*n* = 5; 2%); and a spectrum of other hematologic and non-hematologic conditions with less than five incident cases, including large granular lymphocyte and natural killer cell disorders, chronic myelomonocytic leukemia, hemophagocytic lymphohistiocytosis, anemia of chronic disease, bone marrow failure syndrome, and fungal or viral infection including human immunodeficiency virus and cytomegalovirus; in 12 (4%) cases, no overt associated condition was evident and the cases were accordingly assigned “idiopathic” EMH (further elaborated below). The most frequently involved sites included the spleen (*n* = 164; 53%), liver (*n* = 78; 25%), lymph nodes (*n* = 20; 6%), and the para-spinal region (*n* = 16; 5%) (Table 1). Other sites with lower number of incident cases included the pre-sacral, retroperitoneal, and mediastinal regions, the skull, maxillary region, the skin, kidneys, adrenal tissue, thyroid gland, ovaries, lung, heart, pleura, and pericardium.

**Table 1:** Associated conditions and involved sites among 309 cases of extramedullary hematopoiesis in the absence of myeloproliferative neoplasms

Associated conditions	Involved sites									
	Spleen	Liver	Lymph nodes	Para-spinal region	Retroperitoneal region	Pre-sacral region	Lung	Heart	Mediastinal region	Other sites
All patients (*n* = 309)	164	78	20	16	5	7	8	3	2	34
Myelodysplastic syndromes (*n* = 41)	23	8	4	1	1	0	2	0	0	6
Acute myeloid leukemia (*n* = 28)	12	8	3	1	0	0	1	1	0	6
Hemolytic anemia (*n* = 24)	22	2	0	0	1	0	0	0	0	0
Thalassemia (*n* = 22)	11	4	0	4	1	1	1	0	1	4
Non-Hodgkin’s lymphoma (*n* = 19)	15	2	0	0	0	0	0	0	0	2
Immune thrombocytopenic purpura (*n* = 17)	15	1	2	0	0	0	0	0	0	0
Metastatic cancer (*n* = 17)	3	7	3	1	0	0	1	0	0	3
Plasma cell neoplasms (*n* = 12)	4	5	1	0	0	1	0	0	0	2
Hereditary spherocytosis (*n* = 8)	5	0	0	2	0	1	0	0	0	0
Cirrhosis (*n* = 7)	2	5	0	0	0	0	0	0	0	0
Acute lymphoblastic leukemia (*n* = 6)	3	2	1	0	0	0	0	0	0	0
Chronic lymphocytic leukemia (*n* = 6)	2	1	1	2	0	0	0	0	0	0
Hodgkin’s lymphoma (*n* = 5)	2	3	1	0	0	0	1	0	0	1
“Idiopathic” EMH (*n* = 12)	2	0	0	3	1	4	2	0	0	0
Others (*n* = 93)	43	30	4	2	1	0	0	2	1	10

A diagnosis of “idiopathic” EMH was established in 12 (4%) patients (median age 71 years, range 23–78; 50% females). Most of these cases presented with nonspecific symptoms including abdominal and back pain and the EMH was an incidental discovery (Table 2); 1 patient with splenic EMH presented with fever of unidentified origin (FUO). Involved sites in the 12 patients with idiopathic EMH included 4 pre-sacral, 3 para-spinal, 2 spleen, and 1 each retroperitoneal, pleural-based chest mass, and right upper lobe lung mass. All idiopathic EMH cases were evaluated with imaging studies and diagnosis was subsequently confirmed by pathology review. Past medical history was non-contributory. Complete blood counts were normal in 7 cases, showed anemia in 4 cases, and were not available in 1 case (Table 2). The median follow-up time since the discovery of idiopathic EMH was 7 years (range 2–20). None of the patients with idiopathic EMH showed evidence of any malignancy, including MPN or other hematologic disorders, either at presentation or during follow-up. Nine (75%) of the 12 patients with idiopathic EMH were managed conservatively; the 2 patients with splenic EMH underwent splenectomy and 1 patient had surgical excision of the EMH mass in order to prevent compression of the ureter. The FUO in the patient with splenic EMH resolved with splenectomy.

**Table 2:** Characteristics of 12 consecutive patients with idiopathic EMH

Age/sex	Presentation	Diagnostic procedures	Involved sites	Hemoglobin, g/dl	Leukocyte count, ×10^9^/l	Platelet count, ×10^9^/l	Management	Follow-up since discovery of EMH (years)
71/F	Abdominal pain	MRI, FNA	Pre-sacral mass	13.4	4.4	320	Conservative	9
62/M	Interstitial pneumonia	Wedge biopsies	Right upper lobe lung mass	N/A	N/A	N/A	Conservative	4
23/M	FUO	PET, splenectomy	Splenomegaly	11.1	6	252	Surgical excision	9
78/F	Gallstone evaluation	MRI, FNAB	Pre-sacral mass	14.1	6.1	157	Conservative	7
72/M	Sacral chordoma	CT, FNAB	Pleural-based chest mass	11.4	4.7	131	Conservative	4
50/M	Back pain	CT, FNAB	Left retroperitoneal mass	15.4	11.9	310	Surgical excision	2
72/F	Recurrent UTI	MRI, FNAB	Pre-sacral mass	9.3^a^	10.5	431	Conservative	5
58/F	Para-spinal mass	PET, FNA	Para-spinal mass	10	7.5	295	Conservative	7
71/F	Dyspnea	CT, FNA	Para-spinal mass	14.2	9.6	222	Conservative	4
76/F	Abdominal pain	CT, FNA	Pre-sacral mass	14.2	5.8	244	Conservative	10
61/F	Splenomegaly	CT, splenectomy	Splenomegaly	16.5	7.6	187	Surgical excision	17
70/F	Back pain	CT, FNA	Para-spinal mass	14.8	7.8	171	Conservative	20

*EMH* extramedullary hematopoiesis, *FNA* fine needle aspiration, *FNAB* fine needle aspiration and biopsy, *FUO* fever of unidentified origin, *MRI* magnetic resonance imaging, *N/A* not available, *PET* positron emission tomography, *CT* computed tomography, *UTI* urinary tract infection

^a^Patient was confirmed to have iron deficiency anemia and was successfully treated with iron supplement

We present the largest experience in EMH without associated MPN, in adults. We confirm the spleen and liver being by far the most frequent organs involved; MDS, AML, hemolytic anemia, thalassemia, NHL, ITP, metastatic cancer, and plasma cell neoplasms constituted the most frequent associated conditions. Considering the lack of information, we were particularly interested in “idiopathic” EMH and its natural history. We identified 12 cases of idiopathic EMH, which often represented an incidental discovery during evaluation of unrelated symptoms. None of the patients with idiopathic EMH harbored occult malignancies or subsequently developed MPN or other myeloid malignancies. Accordingly, our observations do not support undertaking extensive investigations targeting MPN or other malignancies in idiopathic EMH and simple monitoring might be adequate.
